# TLR Agonist Augments Prophylactic Potential of Acid Inducible Antigen *Rv3203* against *Mycobacterium tuberculosis* H37Rv in Experimental Animals

**DOI:** 10.1371/journal.pone.0152240

**Published:** 2016-03-29

**Authors:** Owais Mohammad, Jagdeep Kaur, Gurpreet Singh, Syed Mohd Faisal, Asim Azhar, Mohd Ahmar Rauf, Umesh Dutt Gupta, Pushpa Gupta, Rahul Pal, Swaleha Zubair

**Affiliations:** 1 Molecular Immunology Laboratory, Interdisciplinary Biotechnology Unit, Aligarh Muslim University, Aligarh, India; 2 Department of Biotechnology, Panjab University, Chandigarh, India; 3 National JALMA Institute for Leprosy & other Mycobacterial Diseases, Tajganj, Agra, India; 4 National Institute of Immunology, Aruna Asaf Ali Marg, New Delhi, India; 5 Women’s College, Aligarh Muslim University, Aligarh, India; Colorado State University, UNITED STATES

## Abstract

In general, the members of Lip gene family of *Mycobacterium tuberculosis* evoke strong immune response in the host. Keeping this fact into consideration, we investigated role of Rv3203, a cell wall associated protein with lipolytic activity, in imparting protection against experimental murine tuberculosis. The data of the present study suggested that archaeosome encapsulated Rv3203 induce strong lymphocyte proliferation, up-regulated Th-1 biased cytokines profile, increased expression of co-stimulatory markers on both antigen presenting cells and T lymphocytes. The immuno-prophylactic response was further modulated by exposure of the animals to zymosan, a TLR2/6 agonist, prior to immunization with archaeosome encapsulated Rv3203. Interestingly, pre-treatment of experimental animals with zymosan boosted strong immunological memory as compared to archaeosome encapsulated Rv3203 as well as BCG vaccine. We conclude that priming of immunized animal with TLR agonist followed by immunization with archaeosomes encapsulated Rv3203 offer substantial protection against tuberculosis infection and could be a potential subunit vaccine based prophylactic strategy.

## Introduction

The pathogen *Mycobacterium tuberculosis* (*M*. *tuberculosis*), the etiologic agent of human tuberculosis (TB), has been estimated to inflict around 9.6 million people (5.4 million men, 3.2 million women and 1.0 million children) world-wide up to the year 2014 [1–2]. Underlying these statistics is an emerging epidemic of multidrug-resistant tuberculosis (MDR-TB) and extremely drug resistance TB (XDR-TB) [1–3]. The extraordinary potential of *M*. *tuberculosis* to subsist, during the hostile intracellular abode, in macrophages is generally attributed to its ability to modulate host immune responses in its favour [4]. In fact, the pathogen has acquired distinctive ability to subvert fully functioning innate and acquired immune system of the host. There has been a global effort to solve intricacies of the complex interaction between the M. tuberculosis and the host, as pathogen can also shift into a dormant non-replicating status causing a latent TB infection [5].

While immunization plays a key role in tuberculosis control programs, the success rate has been limited due to lack of efficacious vaccine [6]. The problem is further complicated by the variable efficacy of immunizations with Mycobacterium bovis bacillus Calmette-Gue´rin (BCG), the only available vaccine against tuberculosis [7, 8]. Not on a very positive note, BCG, in general, fails to induce herd immunity in a population due to limited efficacy, and also remains unsuccessful to impart long lasting memory response in the host [9]. Of late, it has been observed that BCG often fails in preventing reactivation of latent bacterium [10]. Keeping into consideration the wide spread dissemination of this important disease, it is urgent to search for novel TB vaccines and alternate immunization strategies. Interestingly, it has been found that proteins such as Ag-85, MPT-64, MPB-70, culture filtrate protein-10 (CFP-10) and early secreted antigenic target-6 (ESAT-6), are promising subunit candidates for vaccination against TB, however not as efficacious as BCG [10–14].

*In silico* analysis suggests that *M*. *tuberculosis* genome contains nearly 250 genes encoding putative enzymes involved in lipid metabolism [15]. In fact, most of these enzymes play crucial role in long term survival of *M*. *tuberculosis* in the host macrophages [15]. Inside host’s macrophage, bacteria start accumulating lipid in their cytoplasm to endure dormant state. The lipid droplets serve as carbon and energy source for prolonged survival in the host macrophages which led to an active *Mycobacterial* infection [16]. Besides lipids, lipolytic enzymes too play crucial role in the life cycle, survival and virulence of *M*. *tuberculosis* [17]. A Lip gene family in *M*. *tuberculosis* has been reported to contain 24 putative lipase/esterase (LipC to LipZ) based on the presence of the consensus sequence GXSXG characteristic of members of the α/β hydrolase-fold family [17].

It has been reported that stress-associated membrane bound antigens are potential TB vaccine candidates [18, 19]. We recently demonstrated that Rv3203 (LipV), an acid inducible protein, is a membrane bound protein [20]. Keeping this fact into consideration, we evaluated Rv3203 for its potential to activate both humoral as well as cell mediated components of the host immune system. In the next set of study, archaeosomes entrapped Rv3203 formulation was appraised for its protective efficacy against *M*. *tuberculosis* infection in mice. Zymosan, a TLR2 agonist, has been reported to be a potent adjuvant in stimulating cell mediated immune responses in the host [21]. Zymosan activates macrophages via TLR2 in collaboration with TLR6 which further modulates the immune response against the pathogens [22]. Zymosan has been reported to enhance DC maturation *in vitro* and stimulate production of both inflammatory and type 1 cytokines in the host [23]. Therefore in the present study we explored its capacity to collaborate with novel archaeosome based antigen delivery system to activate host immune system.

## Materials and Methods

### Reagents

Mycobacterium culture medium viz. Middlebrook 7H11 medium, albumin, dextrose, oleic acid, Middlebrook 7H9 broth and catalase etc. were purchased from Difco laboratories (Michigan, USA). pET expression vectors were obtained from Novagen (Darmstadt, Germany). Oligonucleotides were synthesized by BIO Serve (Hyderabad, India). Nickel (Ni/NTA) nitrilo acetic acid metal-affinity chromatography matrix, plasmid miniprep kit and PCR reagents were purchased from Qiagen. The rest of the reagents were of analytical grade of purity and sourced locally.

### Antibodies

Fluorescein isothiocyanate (FITC)–conjugated rabbit anti mouse CD4 and CD8, PerCP conjugated rabbit anti mouse CD62L (MEL-14), phycoerythrin (PE)-conjugated rabbit anti mouse CD44 (IM7), anti-CD80 (B7-1) and anti-CD86 (GL1) were obtained from BD-biosciences. Commercial available kits were used for estimation of various cytokines in plasma as well as culture supernatants.

### Mice

We used 6 to 8 weeks old female *Balb/c* mice procured from National JALMA institute for leprosy and other mycobacterial diseases, Agra, India for various immunological as well as immunoprophylactic studies.

### Ethics Statement

All experimental procedures involving animals were approved by the Institutional Animal Ethics Committees of National JALMA institute for leprosy and other mycobacterial diseases, Tajganj, Agra, Uttar Pradesh, India. All the animal’s studies were performed under BSL-3 animal facilities, in accordance with CPCSEA (Committee for the purpose of control and supervision of experiments on animals, Govt. of India) norms. The study protocol was approved by CPCSEA, Govt of India (332/CPCSEA). All animals were observed for signs of illness, weight loss, injury, or abnormal behaviour by animal house staff twice on week days and only once on weekends. Humane painless handling of all experimental animals includes proper maintenance of living conditions and minimization of distress. The CPCSEA mandates, as formulated by institutional ethical committee, were considered throughout the experimental set up while handling the animals to minimize suffering of animals. Experimental animals were frequently managed and cared throughout the commencement of the study following acceptable standard mandates as specified by ethical committee approved by CPCSEA, Govt of India.

The animals survived after conclusion of the experiment were euthanized following humane endpoint mandates of the institutional animal ethics committee (CPCSEA, Govt of India). The euthanized animals were autoclaved followed by incineration as per the SOPs of BSL-3 lab facilities of NJIL & OMD, Agra (INDIA).

### Bacterial strains

Both *M*. *tuberculosis* H37Rv strains and *M*. *bovis* BCG (Danish) were kind gift from Director, NJIL & OMD, Agra. *M*. *tuberculosis* was grown in Middlebrook 7H9 broth supplemented with OADC and also contained 0.2% glycerol and 0.05% Tween-80 [24].

### Cloning, Expression, Purification and Refolding of Rv3203

The coding region of *Rv3203* from *M*. *tuberculosis* H37Rv was cloned, expressed and purified as described earlier [20]. Briefly, the gene encoding Rv3203 was PCR amplified using set of primer and ligated in pQE30 vector. The ligated product was transformed in *Escherichia coli* (*E*. *coli*) M15 cells and screened on LB plates containing ampicillin (100μg/ml) and kanamycin (30μg/ml). For expression of recombinant *Rv3203*, the *E*. *coli* M15 cells containing pQE30-*Rv3203* plasmid, were grown on LB containing ampicillin (100μg/ml) and kanamycin (30μg/ml). The cells were induced at A_600_ = 0.6 with final concentration of 0.5mM IPTG. After 3 h of induction at 37°C, cells were harvested. The recombinant *Rv3203* purified from inclusion bodies in urea denatured form by Ni-NTA column chromatography. The purified *Rv3203* was analyzed on 12% SDS-PAGE.

### Production of rRv3203 specific polyclonal antibodies

Purified protein *Rv3203* was used to raise polyclonal antibodies in rabbit. Purified protein (0.5mg) was emulsified with complete Freund’s adjuvants and injected subcutaneously. Later, three booster doses were given each at the intervals of 15 days, with purified Rv3203 protein emulsified with incomplete adjuvants. Five days after the last booster dose blood was collected and serum was isolated. The presence of antibody in serum against *Rv3203* was confirmed by ELISA and Western blotting.

### Culture, subcellular fractionation of *M*. *tuberculosis* H_37_Rv and Western blot analysis

H_37_Rv was grown in Middlebrook 7H9 broth base (HiMedia Laboratories Pvt. Ltd. India) supplemented with 1% glycerol and 0.05% Tween-20. An additional 2% (v/v) growth supplement OADC (BBL) was added. Details of the protocols used are provided in S1 File.

### Development and characterization of archaeosomes based antigen delivery system

The membrane lipids isolated from *H*. *Salinarum* were used for preparation of archaeosomes following the method as standardized in our lab [25]. Details of the experimental procedure used are provided in the methods section of the S1 File.

### Preparation of Escheriosomes

The zymosan bearing liposomes were prepared using *E*. *coli* lipid essentially by following the published procedure as standardized in our lab [26]. The details of the experimental protocol are provided in the methods section of S1 File.

### Immunization schedule

The experimental animals were immunized with various in-house developed archaeosome encapsulated formulations of Rv3203 antigen. The formulations were administered at the base of the tail (lower abdominal region) of the experimental animals. Each injection corresponded to 25μg of Rv3203, keeping final volume up to 100 μl of the vehicle (lipid concentration in range of 1.5–20 μg per dose). One group of animals was pre-immunized with escheriosome encapsulated zymosan (50μg/animal) for three consecutive days viz. day–3; day–2 and day -1 prior to immunization with archaeosome encapsulated Rv3203 (day zero). The booster dosages were given on day 21 with matching formulation of antigen using the same route of administration.

### Immunization studies

The details of the protocols for T cells isolation from spleens, antibody isotyping, lymphocyte proliferation, cytokine assay and FACS analysis for the expression of cell surface markers are described in S1 File.

Animals were, humanely, anaesthetized with the combination of 100 mg/kg body weight of ketamine and 10mg/kg body weight of xylazine via intra-peritoneal route. The deeply sedated animals were euthanized by cervical dislocation to perform immunological, histopathological and determination of bacterial burden in their vital organs.

### Determination of bacterial burden in vital organs after challenge with Mycobacterial infection

The animals were divided in various experimental groups as specified in the experimental protocol. Subsequently, animals were challenged with *M*. *tuberculosis* H_37_Rv strain through aerosol route. Briefly, bacterial suspension (5×10^7^/ml) was added to the Aerosol generator device (Glas-col, USA) to inoculate each mouse with approx. 100 bacilli. Prophylactic efficacy of various in-house developed vaccines was assessed on the basis of residual *Mycobacterial* load in lungs and spleens of experimental murine tuberculosis. The follow-up study involves determination of bacterial load in the vital organs of the experimental animals (three animals from each group) on four and eight weeks post challenge with infection. Serially diluted homogenates of the various organs were plated onto 7H11 agar plates supplemented with OADC to count the viable colony-forming units in the organs of the experimental animal. In case of BCG (Danish) immunized group, thiophene carboxylic hydrazide (2mg/ml of concentration) was supplemented to prevent BCG growth and incubated for 3 weeks at 37°C. After stipulated time period, colonies were counted to assess the *Mycobacterial* load.

### Statistical analysis

Statistical significance among experimental groups were analyzed by one way ANOVA (Holm-Sidak method) as well as Student’s t test using Sigma-Plot version 10.0 and 11.0 software. Differences with a *p* value (p≤0.05) were considered statistically significant.

## Results

### Purification and refolding of Rv3203

**Fig 1:**
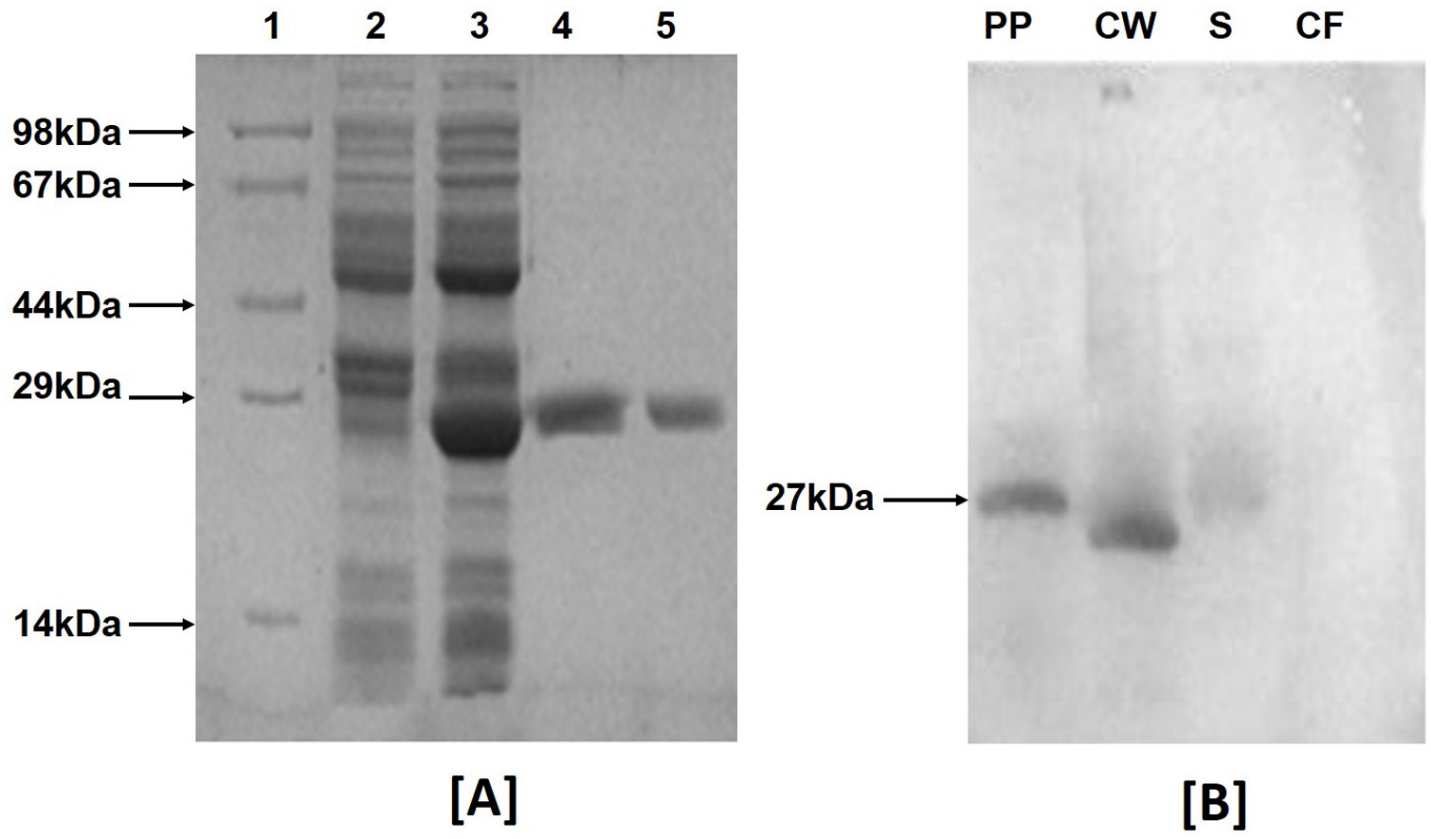
(A) SDS-PAGE analysis of purified Rv3203 protein: Lane 1, protein marker; lane 2, culture pellet uninduced; lane 3, culture pellet induced with IPTG; lanes 4–5, Rv3203 purified protein. (B) Western blot analysis for localization of *Rv3203* in *M*. *tuberculosis* sub-cellular compartments. lane 1; *rRv3203* purified protein (PP), lane 2; cell wall proteins (CW), lane 3; cytosolic proteins (S), lane 4; culture filtrate proteins (CF). Data represent one of the three experiments.

The *Rv3203* gene from *M*. *tuberculosis* H37Rv was PCR amplified, cloned in pQE30 vector and transformed in *E*. *coli* M15. The recombinant Rv3203 was expressed as N-terminal His-tagged fusion protein and isolated as inclusions bodies. The recombinant protein was purified by affinity chromatography on Ni-NTA column. The homogeneity of recombinant Rv3203 was established on the basis of single band in SDS-PAGE (Fig 1A). The molecular weight of the recombinant Rv3203 (fused form) was found out to be around 27kDa.

### Immuno-localization of *Rv3203*

Anti-rRv3203 antibody recognized purified Rv3203 as well as native Rv3203 protein in cell wall fraction of *M*. *tuberculosis* H_37_Rv. The immuno-localization result indicated that Rv3203 is a cell wall associated protein (Fig 1B). No trace of protein was observed in the culture filtrate or the cytosol fraction. The purified protein demonstrated higher molecular weight due to presence of His tag.

### Enhanced Th1 Cytokine induction in the immunized mice

**Fig 2:**
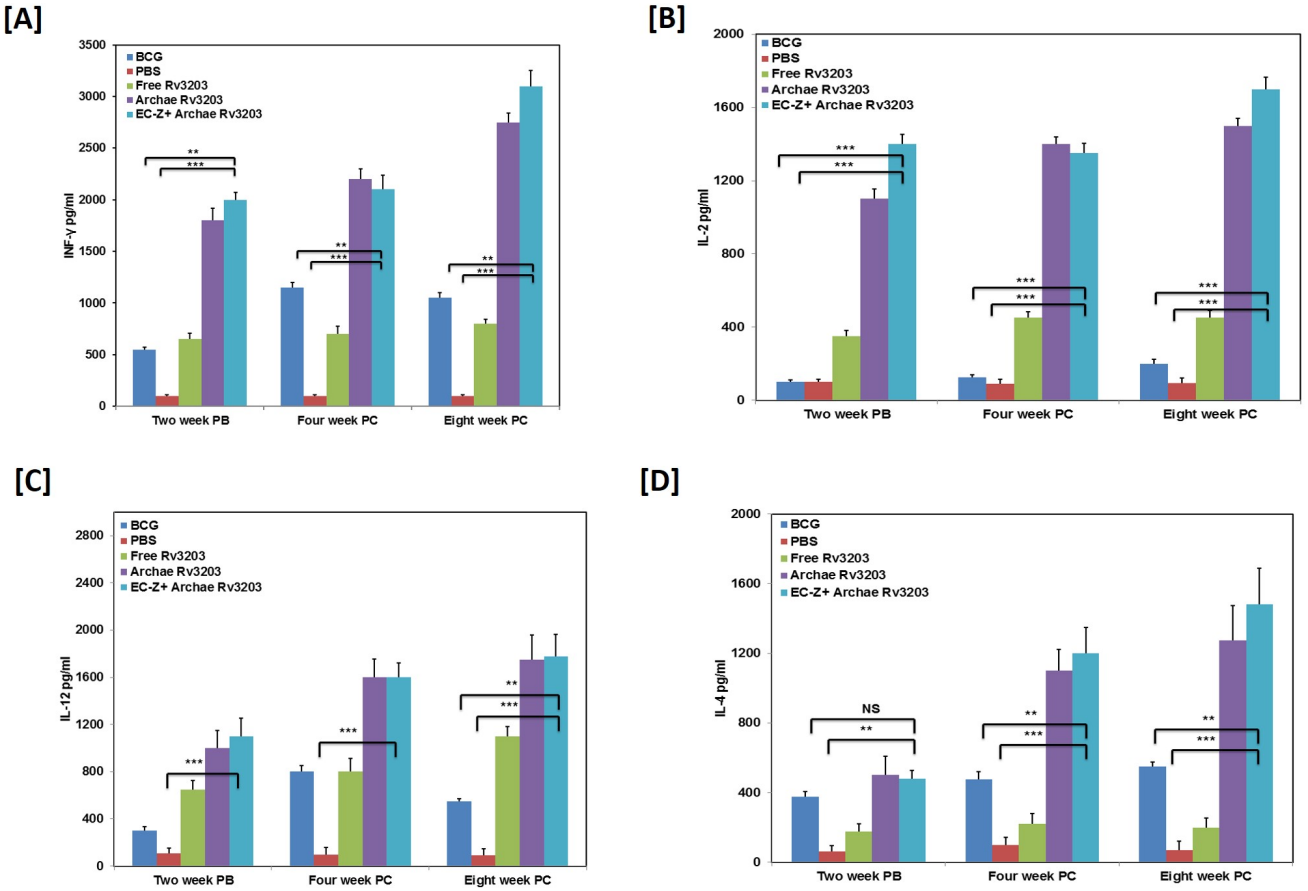
Archaeosome encapsulated Rv3203 has bias to induce type I cytokine expression in the immunized mice. The level of both Type I/Type II cytokines were determined in the splenocyte culture supernatant belonging to various immunized groups at various time points; (A) IFN-γ, (B) IL-2, (C) IL-12, (D) IL-4. (PB- post booster, PC-post challenge).Confirmation of Th1/Th2 polarization expression upon immunization with archaeosome encapsulated *Rv3203*. Data represent mean ± SD from three independent experiments. Statistical significance was determined as described in materials and methods. p≤0.05, p<0.01, p<0.001 and p>0.05 represented as (*), (**), (***) and (NS) respectively.

The immunogenic properties of a novel vaccine formulation is ought to be assessed on the basis of its potential to skew Th1/Th2 cytokine profile bias in the experimental animals. In general, various forms of Rv3203 based vaccine evoked robust T cell proliferation in the immunized animals in dose dependant manner (S1 Fig). Upon further evaluation of immunological outcome of novel Rv3203 based vaccine, we observed Th1 polarization in the immunized animals that were vaccinated with archae-Rv3203 as compared to the free form of Rv3203 and BCG (Fig 2A, 2B and 2C). There was significant up-regulation in the expression of both IL-12 and IFN-γ in the animals that were immunized with archae-Rv3203. The Th1 immune responses were more prominent in the group of animals that were pre-exposed to TLR agonist zymosan (EC-Z + archae-Rv3203).

The immunization protocol did not induce IL-4 up-regulation post immunization, however, its level increased post challenge with infection (Fig 2D). No significant expression of either Th1 or Th2 cytokines was observed in control group of animals (PBS immunization).

### Archaeosome-encapsulated antigen upregulated the expression of co-stimulatory molecules

**Fig 3:**
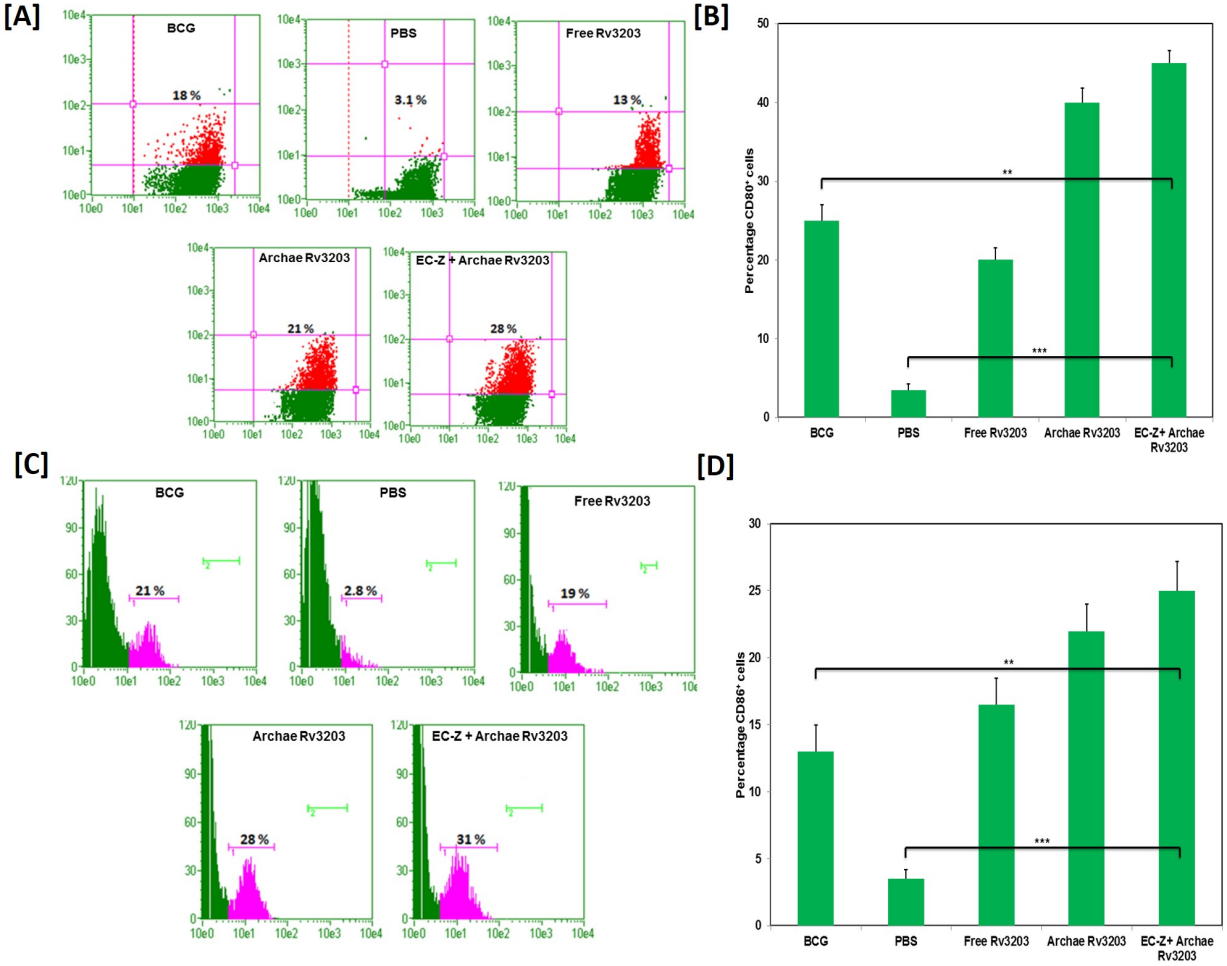
Archaeosome encapsulated Rv3203 up regulates expression of co-stimulatory molecules CD80^+^ and CD86^+^ on antigen presenting cells. The target cells were analysed for presence of CD80+/CD86^+^marker on their surface using flow cytometry (A)& (C) respectively. While (B) and (D) respectively represented the bar form of the expression of CD80+/CD86^+^ molecules on the surface of target cells. Statistical significance was determined as described in materials and methods. p≤0.05, p<0.01, p<0.001 and p>0.05 represented as (*), (**), (***) and (NS) respectively.

The expression profile of co-stimulatory markers was analysed on antigen presenting cells isolated from various group of immunized mice. Interestingly, immunization protocol involving archae-Rv3203 formulation was successful in up-regulating expression of CD80 (B7-1) and CD86 (B7-2) co-stimulatory molecules when compared with BCG and free form of Rv3203, on the 8^th^ week of post challenge with *M*. *tuberculosis* infection (Fig 3). The expression level of CD80 (B7-1) and CD86 (B7-2) was more pronounced in EC-Z + archae-Rv3203 as compared to archae-Rv3203 (no-zymosan) group of animals.

### Archaeosome-encapsulated Rv3203 evoked a strong memory response

**Fig 4:**
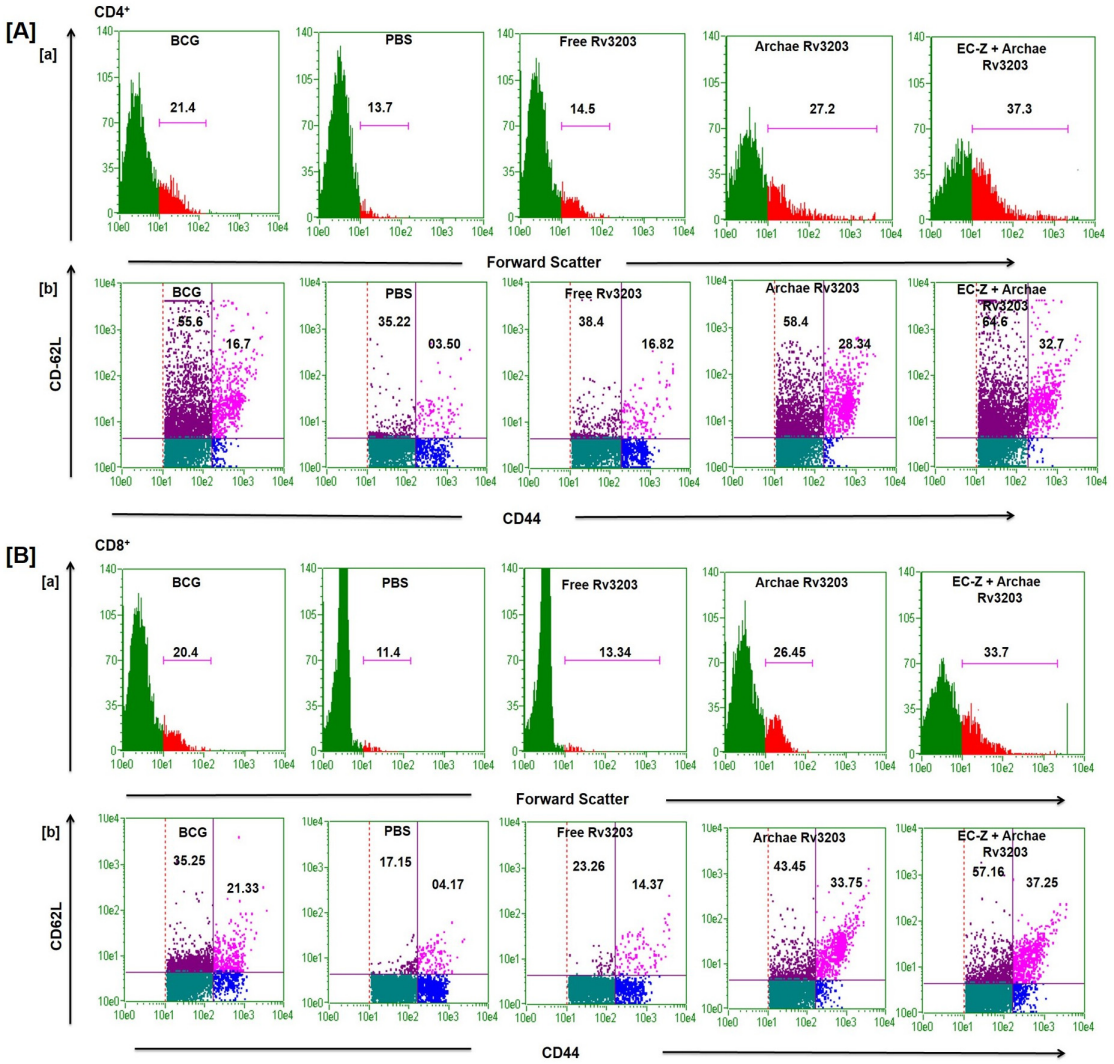
Immunization of mice with archae-Rv3203 augments long-lasting cell memory response. Splenocytes were isolated from various immunized groups after 8 weeks post challenge and analyzed for the presence of CD44^high^ CD62L^low/high^ on gated CD4+ and CD8^+^T cells by FACS (A) and (B) respectively. Data represent mean ± SD from three independent experiments. Statistical significance was determined as described in materials and methods. p≤0.05, p<0.01, p<0.001 and p>0.05 represented as (*), (**), (***) and (NS) respectively.

After 8^th^ week post challenge, both CD4^+^ and CD8^+^ T lymphocytes were isolated from various immunized mice groups. It has been observed that population of CD4^+^ and CD8^+^ T lymphocytes having central memory marker (CD44^high^ and CD62L^high^) and effector memory marker (CD44^high^ and CD62L^low^) on gated population of CD4^+^ and CD8^+^ cells got increased significantly in animal immunized with archae-*Rv3203* and EC-Z+ *Rv3203* when compared to BCG and free Rv3203 as shown in Fig 4. Interestingly, pre-immunization with TLR agonist induced further increase in the population of CD4^+^/CD8^+^ T-lymphocytes with central and effector memory markers. The animals immunized with free form of antigen could not evoke significant T cell population with memory markers.

### Animal protection studies

**Fig 5:**
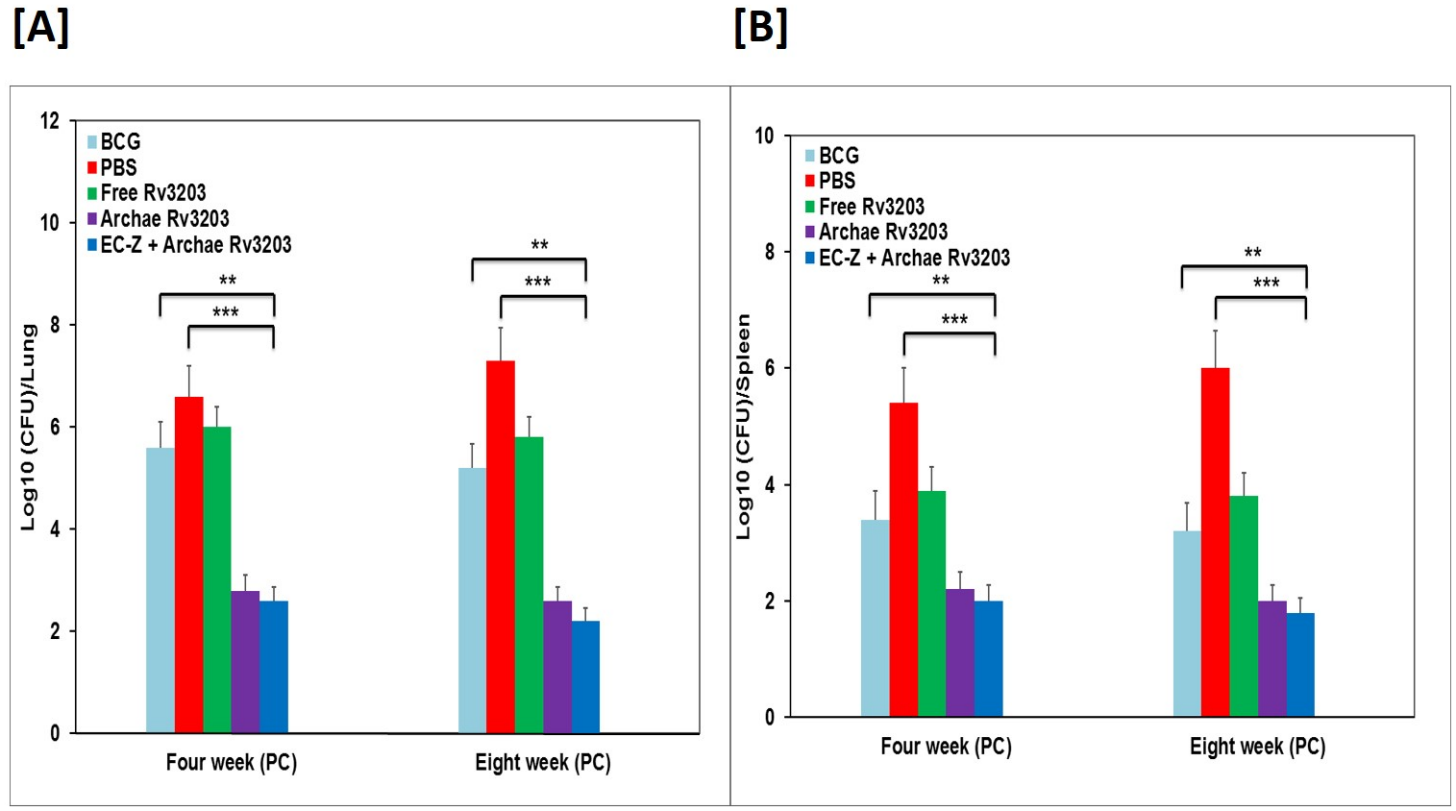
Archaeosomes encapsulated Rv3203 imparts enhance protection against *M*. *tuberculosis* infection in experimental BALB/c mice. Residual bacterial burden in Lungs (A) and Spleens (B), of mice belonging to various immunized groups of experimental animals. CFU at 4 weeks and 8 weeks post challenge following method as described in the materials and methods section of the manuscript text. Statistical significance was determined as described in materials and methods. p≤0.05, p<0.01, p<0.001 and p>0.05 represented as (*), (**), (***) and (NS) respectively.

The vaccine potential of various in-house developed formulations was assessed on the basis of their ability to eliminate *Mycobacterial* burden in lungs and spleen of the experimental murine animals. As enumerated on 4th week post challenge, archae-Rv3203 vaccine formulation exhibited superior protection and caused significant reduction in *Mycobacterial* burden as compared to both free form of Rv3203 as well as BCG (**p<0.01). Animals vaccinated with archae-Rv3203 in combination with EC-Z were found to be better protected when compared to archae-Rv3203 combination and exhibited a significant reduction in *Mycobacterial* burden in various organs (Fig 5). However, on 8th week post challenge, the *Mycobacterial* load in the animal groups immunized with archae-Rv3203 alone or EC-Z + archae-Rv3203 was found out to be of the same order (Fig 5).

## Discussion

Lipases are crucial for maintaining virulence and subsistence of *M*. *tuberculosis* in the host. In general, lipolytic enzymes carry out important physiological functions and participate in the extraordinary capacity of *M*. *tuberculosis* to subjugate hostile intracellular abode in the infected host [27–29]. It has been reported that Mycobacterial genes involved in lipid metabolism are up-regulated during latent phase of infection [30]. Besides RD antigens, BCG has been reported not to share dormant state antigens with *M*. *tuberculosis*, leading us to believe that lipases induced during latent phase, can be considered as potent targets for developing future potential vaccine candidate [31, 32].

Employing immuno-informatics analysis, Rv3203 was predicted as a cytosolic protein; however, the immune-localization studies performed with specific polyclonal antibodies suggested that the enzyme is mainly confined to the cell wall fraction of *M*. *tuberculosis*. The membrane bound proteins are reported to be accessible to interact with host immune system more vigorously [18]. Keeping this fact into consideration, Rv3203 was selected as contender protein to study its potential as candidate vaccine. We have also investigated its efficacy to induce protective immunity in the immunized mice against experimental tuberculosis.

Recently, archaeosomes based antigen delivery system has been shown to induce strong cell mediated as well as humoral response against entrapped antigens in the host [33, 34]. Considering strong immune-adjuvant potential of archaeosome, we performed an extended study to correlate host immune responsiveness against a recombinant rRv3203 protein bearing archaeosome based vaccine. The novel antigen delivery system was also evaluated for its potential to impart long lasting protection against *M*. *tuberculosis*. As archaeosomes mediated activation of the host immune system does not involve TLR mediation, we manipulated an immunization strategy that involved pre-immunization with TLR agonist to further activate host immune system for desirable response [35].

Lymphocyte proliferation assay revealed enhanced T cell proliferation (Rv3203 specific) with both archae-Rv3203 as well as EC-Z+ archae-Rv3203 formulation in the immunized animals (S1 Fig). The lymphocyte proliferation was considerably lesser in the animals immunized with free form of Rv3203. In a nutshell, the formidable capability of both archae-Rv3203 and EC-Z+ archae-Rv3203 formulation to induce a stable and consistent T cell activation (both CD4^+^/CD8^+^) favours its development as a new and effective vaccine candidate (S1 and S2 Figs).

Cytokine profiling further refined the notion that levels of IFN-γ and IL-12 were higher in animals primed with EC-Z+ archae-Rv3203 formulation as well as archae-Rv3203 (with no zymosan pre-treatment) (**p<0.01). Among the two immunization strategies, Th1 biased response was more prominent in animals pre-treated with TLR agonist zymosan when compared to archaeosome based Rv3203 immunization alone or free form of Rv3203. On the other hand, the Th2 cytokine expression was substantially less in the animals immunized with archaeosome based *Rv3203*. Moreover, Th2 cytokines also were not up-regulated in zymosan pre-treated animals (pre challenge studies).

The level of IL-4 increased in the group of mice that was immunized with various combinations such as archae-Rv3203 and EC-Z + archae-Rv3203 post challenge with infection. This can be attributed to the fact that exposure with virulent H_37_Rv (upon challenge) might skew immune response (Th2 type) that favours survival of pathogen in the host. Nevertheless, both archae-Rv3203 and its combination with TLR agonist were still capable of manipulating host immune response to resist pathogen survival.

As demonstrated in S3 Fig, the archae-Rv3203 and EC-Z+ archae-Rv3203 were also successful in inducing higher IgG2a versus IgG1 in the immunized experimental animals at two week PB (post booster). Further the ratios of the two antibody isotypes were increased at post challenge in *M*. *tuberculosis* infection. Whereas in BCG vaccinated experimental animals, the isotype ratio was nearly similar in both PB as well as PC (post challenge) status (S3 Fig). Th1/Th2 dichotomy regulates priming of B cells for production of antibodies with IgG1 or IgG2a phenotype [36–38]. The observed antibody isotyping result indicated that archaeosomes mediated up-regulation of IFN-γ assign B cell for more IgG2a production. The data of both cytokine profiling as well as antibody isotyping suggests that EC-Z+ archae-Rv3203 form of antigen has outstanding potential to evoke Th1 type immune response in the host, essential feature for desirable prophylaxis against *M*. *tuberculosis*.

The zymosan based novel antigen delivery strategy show maximum up-regulation of co-stimulatory molecules on APCs of the immunized animals. This was suitably complemented with development of T cell population with CD44^high^CD62L^high^as well as CD44^high^CD62L^low^phenotype in the animals immunized with archae-Rv3203 and EC-Z+ archae-Rv3203 compared to free Rv3203 and BCG immunized groups. It has been observed that immunization schedule involving combined administration of TLR agonist and archaeosome-Rv3203 ensues in better up-regulation of memory phenotype in Rv3203 specific T cells induced in the host [39]. The central memory response accompanied with CD44^high^CD62L^high^phenotype is considered to be involved in containment of secondary infection [40]. On the other hand, effector memory response accompanied with CD44^high^CD62L^low^ phenotype is reported to be enhanced during chronic infections [41, 42]. The long-lasting memory response envisages archae-Rv3203 and TLR agonist zymosan combination as an effective prophylactic approach that augments efficient interactive competence of archaeosomes with APCs, especially dendritic cells [35]. Importantly, the immune-adjuvant potential of TLR agonist zymosan combined well with archaesome based Rv3203 vaccine to impart significant protection of immunized animals with *M*. *tuberculosis* challenge.

## Conclusion

Finally, we infer that archaeosomes based Rv3203 can successfully confer long lasting and effective protection against experimental murine tuberculosis. Further, the data of the present study also suggest that immune activation as well as protection efficacy can be further enhanced by priming of animals with TLR agonist prior to immunization. Archaeosomes based Rv3203 subunit vaccine candidate in combination with TLR agonist has potential to evoke desirable immune response and elicits *Mycobacterium sps* specific T cells as well as antibodies in the host. Besides tuberculosis, the priming of TLR agonist bearing nano-vaccines may offer effective strategy for elimination of other intracellular pathogens as well.

## Supporting Information

S1 Fig T cell proliferation response in various immunized groups to determine the effect of dose of Rv3203 on proliferation of T lymphocytes; splenocytes, isolated from various groups of immunized *BALB/c* mice at two weeks post booster time point, were co-cultured in the presence of increasing amounts (1.56 to 50 μg) of *Rv3203* Ag in flat-bottomed 96 well plates. After 72 h, [^3^H]-thymidine was added to each well and its incorporation in multiplying cells was measured after 16 h incubation with liquid scintillation counting. The accumulation of ^3^H, thymidine was determined in proliferating cells and denoted in term of counting per minute (CPM) values of stimulated cultures to represent Ag specific stimulation.
(TIF)

S2 Fig Proliferation of *Rv3203* specific T lymphocytes isolated from immunized animals at two week post booster and also at 4 and 8 weeks post challenge upon stimulation with a fixed amount (50μg) of free *Rv3203*. Data represents the mean of three determinants ± S.D. (PB- post booster, PC-post challenge).Statistical significance was determined as described in materials and methods. p<0.01, p<0.001 and p>0.05 represented as (**), (***) and (NS) respectively.
(TIF)

S3 Fig The sera of immunized animals were analysed for the evaluation of IgG2a: IgG1 isotypes by sandwich ELISA method. The data represent mean of three determinants ± S.D. and are representative of two different experiments with similar observation. (PB-post booster, PC-post challenge). Statistical significance was determined as described in materials and methods. p<0.01, p<0.001 and p>0.05 represented as (**), (***) and (NS) respectively.
(TIF)

S1 File Contains Supplemetary Methods and Supplementary Results. Supplementary Methods contains following methods in detailed description (A) Culture, subcellular fractionation of *M*. *tuberculosis* H37Rv and western blot analysis, (B) Development and characterization of archaeosome based vaccine, (C) Preparation of Escheriosomes, (D) Assessment of antibody isotype in sera of experimental immunized animals, (E) Isolation of T cells from spleens of experimental immunized animals, (F) Lymphocyte proliferation assay, (G) Cell culture and cytokine assay: Determination of IFN-γ, IL-4 and IL-12, (H) Determination of cell surface markers expression as revealed by Flow cytometry. Supplementary Results contains following results in detailed description (A) ArchaeRv3203 augment the lymphocyte proliferation, (B) Archaeosome encapsulated Rv3203 evokes predominantly IgG2a and IgG2b type antibodies in the immunized mice.
(DOC)
