# Supplementary figures and images for: TLR Agonist Augments Prophylactic Potential of Acid Inducible Antigen Rv3203 against Mycobacterium tuberculosis H37Rv in Experimental Animals
Source: PLoS One. 2016 Mar 29;11(3):e0152240. doi: 10.1371/journal.pone.0152240 (PMC4811581; doi:10.1371/journal.pone.0152240)

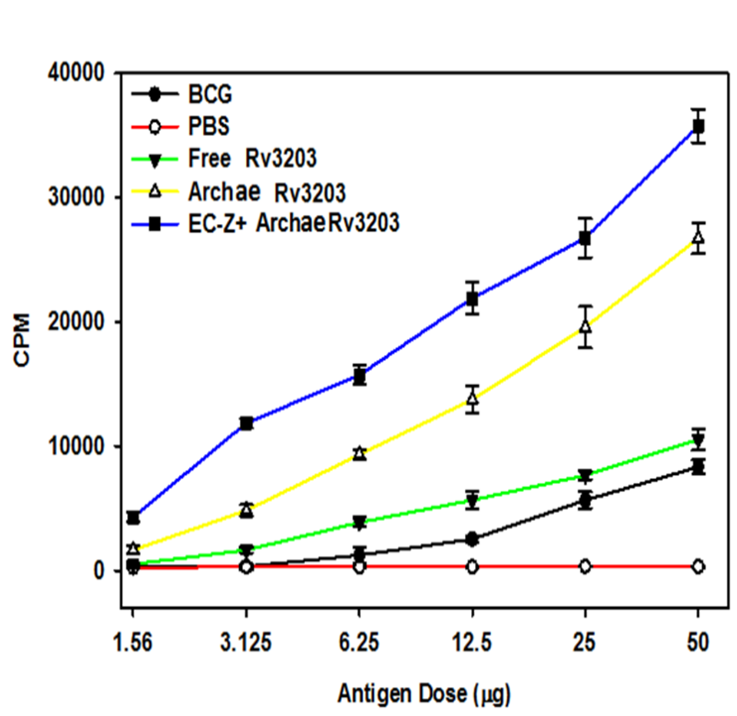

Supplement: S1 Fig — After 72 h, [3H]-thymidine was added to each well and its incorporation in multiplying cells was measured after 16 h incubation with liquid scintillation counting. The accumulation of 3H, thymidine was determined in proliferating cells and denoted in term of counting per minute (CPM) values of stimulated cultures to represent Ag specific stimulation. (TIF) [file pone.0152240.s001.tif]

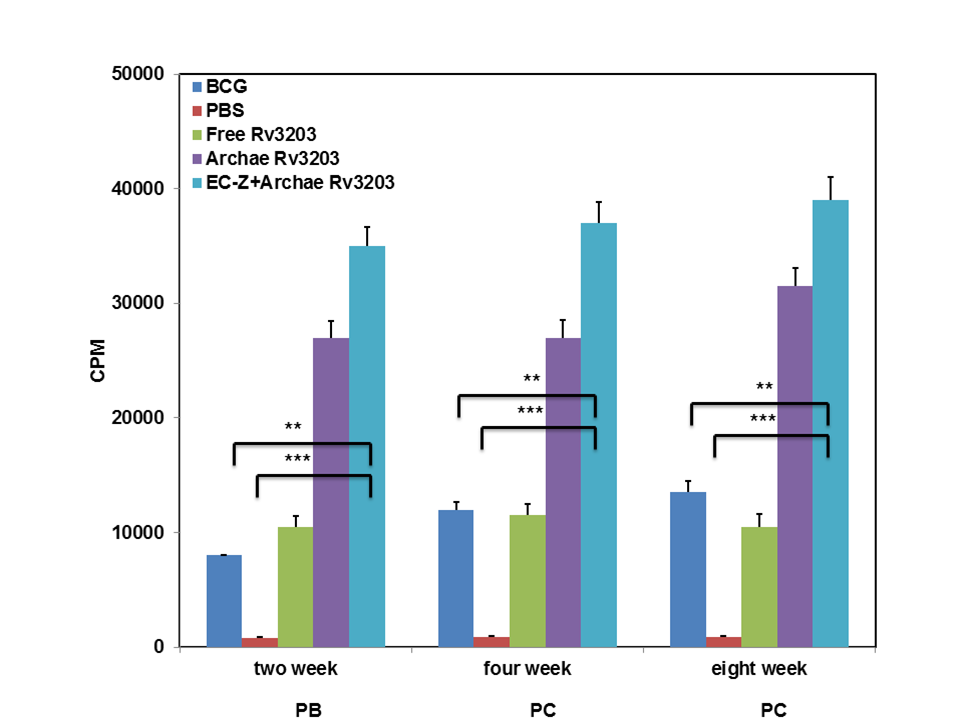

Supplement: S2 Fig — Data represents the mean of three determinants ± S.D. (PB- post booster, PC-post challenge).Statistical significance was determined as described in materials and methods. p<0.01, p<0.001 and p>0.05 represented as (**), (***) and (NS) respectively. (TIF) [file pone.0152240.s002.tif]

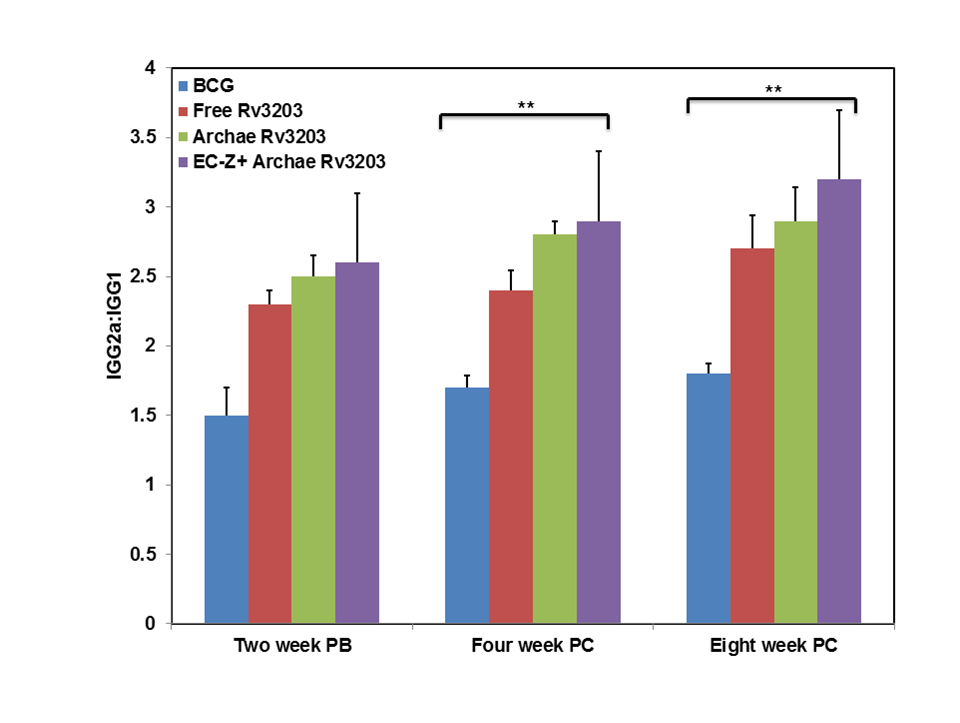

Supplement: S3 Fig — The data represent mean of three determinants ± S.D. and are representative of two different experiments with similar observation. (PB-post booster, PC-post challenge). Statistical significance was determined as described in materials and methods. p<0.01, p<0.001 and p>0.05 represented as (**), (***) and (NS) respectively. (TIF) [file pone.0152240.s003.tif]
