# Supplementary material for: TLR Agonist Augments Prophylactic Potential of Acid Inducible Antigen Rv3203 against Mycobacterium tuberculosis H37Rv in Experimental Animals
Source: PLoS One. 2016 Mar 29;11(3):e0152240. doi: 10.1371/journal.pone.0152240 (PMC4811581; doi:10.1371/journal.pone.0152240)
Supplement: S1 File — Supplementary Methods contains following methods in detailed description (A) Culture, subcellular fractionation of M. tuberculosis H37Rv and western blot analysis, (B) Development and characterization of archaeosome based vaccine, (C) Preparation of Escheriosomes, (D) Assessment of antibody isotype in sera of experimental immunized animals, (E) Isolation of T cells from spleens of experimental immunized animals, (F) Lymphocyte proliferation assay, (G) Cell culture and cytokine assay: Determination of IFN-γ, IL-4 and IL-12, (H) Determination of cell surface markers expression as revealed by Flow cytometry. Supplementary Results contains following results in detailed description (A) ArchaeRv3203 augment the lymphocyte proliferation, (B) Archaeosome encapsulated Rv3203 evokes predominantly IgG2a and IgG2b type antibodies in the immunized mice. (DOC) [file pone.0152240.s004.doc]

**Title:**

TLR agonist augments protection potential of acid inducible antigen *Rv3203* against *Mycobacterium tuberculosis* H37Rv in experimental animals

**Authors:**

Owais Mohammad^†1^, Jagdeep Kaur^†2^, Gurpreet Singh^†2^, Syed Mohd Faisal^1^, Asim Azhar^1^, Mohd Ahmar Rauf^1^, Umesh Dutt Gupta^4^, Pushpa Gupta^4^, Rahul Pal^5^, Swaleha Zubair^6*^

^1^Molecular Immunology Laboratory, Interdisciplinary Biotechnology Unit, Aligarh Muslim University, Aligarh, 202002, India

^2^Department of Biotechnology, Panjab University, BMS Block1, Sector 25, Chandigarh, 160014, India

^3^National JALMA Institute for Leprosy & other Mycobacterial Diseases, Tajganj, Agra, 282006, India

^4^National Institute of Immunology, Aruna Asaf Ali Marg, New Delhi, 110067, India.

^5^Women’s College, Aligarh Muslim University, Aligarh, 202002, India

**Supplementary Methods**

**(A) Culture, subcellular fractionation of *M. tuberculosis* H37Rv and western blot analysis**

H37Rv was grown in middlebrook 7H9 broth base (HiMedia Laboratories Pvt. Ltd. India) supplemented with 1% glycerol and 0.05% Tween-20. An additional 2% (v/v) growth supplement OADC (BBL) was added. Briefly, the culture of *M. tuberculosis* H37Ra (at mid log phase, A_600_ – 1.5) was centrifuged at 10,000*g* for 30 min at 4ºC. Culture supernatant was passed through 0.22-µm pore size filter and the culture filtrate protein fraction (CFP) was lyophilized. Pelleted cells were washed, re-suspended in PBS and lysed by using the probe sonicator (10 x of 60 s cycle with 2 min cooling period between each burst). Lysate was centrifuged twice at 11,000*g* for 5 min at room temperature to remove unbroken cells and insoluble materials. Lysate supernatant was centrifuged at 27,000 *g* for 1h at 4ºC to separate the cytosolic fractions (supernatant) and cell wall fractions (pellet) (1). For western blot analysis, 1µg of purified protein and 3µg of each cell wall (CW), cytosolic (S) and culture filtrate protein (CF), were loaded on 12% SDS-PAGE and transferred to nitrocellulose membrane. After blocking the nitrocellulose membrane with 5% skimmed milk in PBS, the nitrocellulose membrane was incubated with polyclonal antibodies against *Rv3203* (final dilution 1:200) for 2h. After three washes with PBST, the membrane was incubated for 1h with alkaline-phosphatase conjugated anti-rabbit IgG (raised in goat, final dilution 1:5000) and blots were developed by using BCIT/NBS as substrate.

**(B) Development and characterization of archaeosome based vaccine**

To ameliorate potential of candidate vaccine Rv3203, we had developed an archaeosome based antigen delivery system. Archaeosomes were prepared from membrane lipids isolated from *H. salinarium.* Archaeal lipid based dried and reconstituted vesicles were prepared by following the method as standardized in our lab (2). In brief, lipid suspension was prepared by hydrating dry lipid with normal saline and then homogenous population of unilamellar liposomes were obtained through sonication of hydrated lipid in bath sonicator at 4˚C (Power sonics, South Korea). The unilamellar liposomes thus prepared were centrifuged for 10 min at 11,500 ×g at 4˚C to remove large lipid aggregates (3). Finally liposome suspension was mixed with equal volume of protein solution. To increase entrapment efficiency several freeze thaw cycles were executed.

**(C) Preparation of Escheriosomes**

The zymosan bearing liposomes were prepared using *Escherichia coli* lipid essentially by following the published procedure as standardized in our lab (4). Briefly, *E. coli* lipids (total lipid 20 mg) were reduced to thin dry film under N_2_ atmosphere. The film was hydrated, sonicated using bath sonicator for 1 hr at 4˚C. The liposomes thus formed were mixed at this stage with an equal volume of zymosan (30mg/ml stock). The mixture was frozen and thawed (3 cycles) very quickly and then lyophilized. The lyophilized liposomes thus obtained was rehydrated with distilled water (120µl) and finally re-constituted with PBS. The re-constituted liposomes were centrifuged at 14,000×g and the pellet thus obtained was further washed to remove the traces of the un-entrapped solute. The liposomes (given volume) were lysed with 10% Triton X-100 solution (the final concentration of Triton X-100 was maintained 1%) and the amount of zymosan entrapped was estimated by HPLC.

**(D) Assessment of antibody isotype in sera of experimental immunized animals**

To analyze the presence of antigen specific antibodies sera were collected at different time points from various experimental group of animals. Subsequently, their isotypes were analyzed. In brief, antigen were coated for overnight on 96 well microtiter plates in appropriate buffer at 4˚C. After washing, wells were blocked and plates were finally incubated with the standards and sera at 37 °C for 2hr. Goat anti-mouse anti-IgG1 and IgG2a antibodies were added after washing and incubated at 37 °C for 1hr. Further, after designated time secondary reagents i.e. HRP conjugated rabbit anti-goat antibody were added to each plate and absorbance was read at 490 nm with a micro titer ELISA plate reader (Genetix GMB-580).

**(E) Isolation of T cells from spleens of experimental immunized animals**

Various experimental immunized groups were euthanized at PB (post booster) as well as PC (post challenge) with infection. Splenocyte cell suspension was prepared as described previously (5). Briefly, spleens were isolated from experimental animals, macerated and single cell suspension was treated with cold water for the lysis of RBCs. The cell suspension was then centrifuged at 1200g for 10 min and cell pellet was washed twice and re-suspended in RPMI complete media.

**(F) Lymphocyte proliferation assay**

Lymphocyte proliferation assay was performed as described elsewhere (5). In brief, lymphocytes were isolated from the spleens of various groups of immunized mice, and cultured in triplicate at 2×10^5^ cells per well with graded doses (1–50µg/well) of Rv3203 in 200µL of RPMI 1640 medium supplemented with 10% fetal bovine serum in 96-well flat bottom plates. In the next set, splenocytes from various experimental immunized groups were incubated with a known amount of corresponding matching formulations of Rv3203. Splenocytes incubated with the medium alone were used as control. Plates were pulsed with 0.5mCi of [^3^H]-thymidine 3 days after initiation of culture. After 16hr, plates were harvested onto glass-fiber filter mats by the use of Tomtec-Harvester-96 (Tomtec) for liquid scintillation spectroscopy (Wallac-1450 Microbeta Trilux; Perkin Elmer).

**(G) Cell culture and cytokine assay: Determination of IFN-γ, IL-4 and IL-12**

Induction of Th1 & Th2 paradigm by splenocytes upon their co-culture in the presence of various forms of Rv3203 were estimated following published protocol elsewhere (6). Briefly, 96-well ELISA plates were coated overnight with capture antibodies at 4°C. After washing, wells were blocked with 200 µl of assay diluents (BD Biosciences) at 37 °C for 2hr. The plates were finally incubated with the standards and culture supernatants (isolated from cultured splenocytes after 48h to determine its cytokine content) at 37°C for 2hr. After washing, biotinylated polyclonal goat anti-mouse cytokine antibodies (secondary) were added and incubated at 37 °C for 1hr. Further, after washing the plates, streptavidin-HRP conjugate was added to each well and incubated for 1hr at 37°C. Finally, plates were washed and colored complex was developed using 3,3’,5,5’-tetramethylbenzidine substrate and reaction was stopped after 15min using 2N sulphuric acid. The absorbance were read at 490nm with a microtiter plate reader (Genetix GMB-580).

**(H) Determination of cell surface markers expression as revealed by Flow cytometry**

Single cell suspension from spleen was prepared and analyzed on Easycyte Mini (Guava Technologies) flow cytometer as described elsewhere (7). In brief, 1×10^6^cells were washed with FACS buffer and incubated with Fc block (2.4G2) or with FITC/PE/PerCP labeled monoclonal antibodies specific for CD4, CD8, CD44, CD62L, CD80, CD86 and isotype control for 30 min at 4˚C. Data were analyzed using Express-Plus software. The total number of cells of a definite phenotype (CD4^+^CD44^high^CD62L^low/high^, CD8^+^CD44^high^CD62L^low/high^) were calculated by taking the percentage of the gated cell population, as determined by FACS analysis, multiplying them with the total number of cells obtained per mouse, and finally dividing the furnished result by the number of events.

**Supplementary Results**

**(A) ArchaeRv3203 augment the lymphocyte proliferation**

To assess the vaccine potential of archaeRv3203, proliferation of lymphocytes in response to different form of Rv3203 was measured in immunized animals. The lymphocytes were isolated at various time points (post booster and post challenges) from spleen of immunized mice. As shown in **S1 Fig**, proliferation of lymphocytes was observed in dose dependent pattern. In the case of both archreaRv3203 and EC-Z + archaeRv3203, the lymphocyte proliferation rate was significantly higher than free form of antigen and BCG, on increasing the dose of antigen from 1.56μg to 50μg. Mice belongings to negative control (PBS) did not induce considerable proliferation of lymphocytes. Histogram revealed the lymphocyte proliferation response for a fixed dose of antigen (50μg) at different time points, viz, two week post-booster, four and eight week post-challenge with infection. Among various forms of antigens, EC-Z+ archaeRv3203 maintained a higher proliferative response at various time points (post-booster as well as post-challenge), when compared to the free form of antigen and BCG (Post-booster as well as post-challenge) (figure 3B). At 8^th^ week post challenge, lymphocyte proliferation rate for EC-Z+ archaeRv3203 was higher than T cell proliferation rate at 4^th^ week post challenge. The T cell proliferation pattern was same for determination made at two week post booster **(S2 Fig)**.

**(B) Archaeosome encapsulated Rv3203 evokes predominantly IgG2a and IgG2b type antibodies in the immunized mice**

To determine the antibody isotype switching, we evaluated Rv3203 antigen specific IgG1, IgG2a, and IgG2b antibody response in serum of immunized animals at various time points. As shown in **S3 Fig**, the ratio of IgG2a to IgG1 in BCG immunized group at two week post booster, and 4^th^ and 8^th^ week post challenge time points did not increase significantly. The ratio of IgG2a to IgG1 in free Rv3203 immunized animals was observed statistically significant at any time points when compared with BCG. Furthermore, this ratio was further upregulated for archaeRv3203 and EC-Z + archaeRv3203 immunized mice. The isotype data suggest that EC-Z + archaeRv3203 antigen induced better Th1 response in immunized animals when compared to other groups including BCG immunized group of animals.

**References**

1. **Rezwan M1, Lanéelle MA, Sander P, Daffé M.** Breaking down the wall: fractionation of mycobacteria. J Microbiol Methods.2007;**68:**32-39.
2. **Ansari MA, Zubair S, Mahmood A, Gupta P, Khan AA, Gupta UD, Arora A, Owais M.** [RD antigen based nanovaccine imparts long term protection by inducing memory response against experimental murine tuberculosis.](http://www.ncbi.nlm.nih.gov/pubmed/21853054) PLoS One. 2011;**6:**e22889.
3. **Kirby CJ, Gregoriadis G.** [Preparation of liposomes containing factor VIII for oral treatment of haemophilia.](http://www.ncbi.nlm.nih.gov/pubmed/6443849) J Microencapsulation. 1984;**1:**33-45.
4. **Chauhan A, Swaleha Z, Ahmad N, Farazuddin M, Vasco A, Abida M, Mohammad O.** [Escheriosome mediated cytosolic delivery of Candida albicans cytosolic proteins induces enhanced cytotoxic T lymphocyte response and protective immunity.](http://www.ncbi.nlm.nih.gov/pubmed/21645572) Vaccine. 2011;**29:**5424-4533.
5. **Chauhan A, Zubair S, Ahmad N, Ansari SA, Ansari MY, Owais M.** Escheriosome-mediated cytosolic delivery of PLK1-specific siRNA: potential in treatment of liver cancer in BALB/c mice. Nanomedicine. 2014;9(4)**:**407-420.
6. **Darrah PA, Patel DT, De Luca PM, Lindsay RW, Davey DF, Flynn BJ, Hoff ST, Andersen P, Reed SG, Morris SL, Roederer M, Seder RA.** Multifunctional TH1 cells define a correlate of vaccine-mediated protection against Leishmania major. Nat Med. 2007;**13:**843-850.
7. **Zhang Y, Buchholz F, Muyrers JP, Stewart AF.**[A new logic for DNA engineering using recombination in Escherichia coli.](http://www.ncbi.nlm.nih.gov/pubmed/9771703) Nat Genet. 1998;**20:**123-128.
